# Neurocysticercosis Presenting as Status Epilepticus

**DOI:** 10.7759/cureus.88302

**Published:** 2025-07-19

**Authors:** Vaaragie Subramaniam, Jessica Houck DO

**Affiliations:** 1 Emergency Medicine, University of Kentucky, Lexington, USA

**Keywords:** neurocysticercosis, parasitic infections, scolex, seizure, status epilepticus, taenia solium

## Abstract

A 26-year-old healthy female with recent immigration from Nepal presented to the emergency department (ED) after experiencing four to five generalized tonic-clonic seizures at home. Workup in the ED revealed an abnormal calcification of the occipital lobe with vasogenic edema. The patient was given lorazepam and loaded with levetiracetam and dexamethasone. She was admitted to the hospital for new-onset seizures and workup of brain mass. An inpatient MRI revealed a solitary ring-enhancing lesion with punctate calcifications, consistent with a scolex, and blood analyses confirmed the diagnosis of neurocysticercosis. The patient was treated successfully with albendazole and corticosteroids and discharged after four days with no recurrence of seizures to date.

## Introduction

Neurocysticercosis (NCC) is a parasitic infection caused by the helminth, *Taenia solium* (*T. solium*), that forms cysts consisting of parasitic larvae that can invade any tissue in the body, especially the brain. When undiagnosed and untreated, neurocysticercosis can cause seizures (as seen in this patient) as well as increased intracranial pressure and can be fatal in rare circumstances. NCC is the leading cause of acquired epilepsy in developing countries [1,2].

NCC is acquired from ingesting undercooked pork and is most commonly found in developing countries. It is estimated that the total number of people in the world with NCC (both symptomatic and asymptomatic) is 2.56-8.3 million [3]. According to the NIH, it is a rare diagnosis in the United States, with estimates of 0.2 to 0.6 cases per 100,000 of the general population [1]. However, it is important to be aware of the symptoms and clinical findings of NCC because there is an increasing number of immigrant populations who are at higher risk of exposure to *T. solium* and therefore at increased risk of acquiring the disease. Even if the patients don't have a recent history of travel to endemic countries, it still should be on the differential because the mean incubation period of NCC is 3.5 years [4]. 

We present a case of a patient who immigrated from Nepal, presenting to the emergency department with status epilepticus and was diagnosed with neurocysticercosis.

## Case presentation

A 26-year-old female with a past medical history of hypothyroidism presented to the emergency department via ambulance after experiencing multiple generalized tonic-clonic seizures at home. The patient arrived very somnolent and reported experiencing a slight headache for the past day. She had no history of seizures and denied fever, vomiting, recent trauma, changes in vision, or neurological symptoms. She denied any recent vomiting, diarrhea, abdominal pain, weight loss, or change in stool. Social history revealed she immigrated from Nepal two years prior. She denied alcohol or substance abuse. 

She was accompanied by her friend, who was present during the seizure episodes, and he described multiple episodes of generalized tonic-clonic activity with loss of consciousness and tongue biting. The first episode lasted approximately five minutes, and she had four more episodes with five minutes between each episode, where she was somnolent and confused. After the third seizure, the patient began vomiting.

Initial vitals upon arrival to the emergency department, including point of care glucose, were all within normal limits, and the patient was afebrile. Physical exam revealed a somnolent but arousable young female in no acute distress. Neurologic exam was unrevealing, and a small tongue laceration was noted with hemostasis achieved. The differential diagnosis at this point was new-onset epilepsy, brain tumor, intracerebral hemorrhage, encephalitis/meningitis, and cerebral venous sinus thrombosis. Workup in the ED included computed tomography (CT) head without contrast, CT venogram head, chest X-ray, drug abuse screen, blood cultures, urinalysis, pregnancy test, chemistry panel, and complete blood count (CBC).

Shortly after workup was initiated, the patient went on to have another generalized tonic-clonic seizure that required 2mg of intravenous (IV) lorazepam. The patient was loaded with 4 grams of levetiracetam to prevent further seizures.

**Figure 1:**
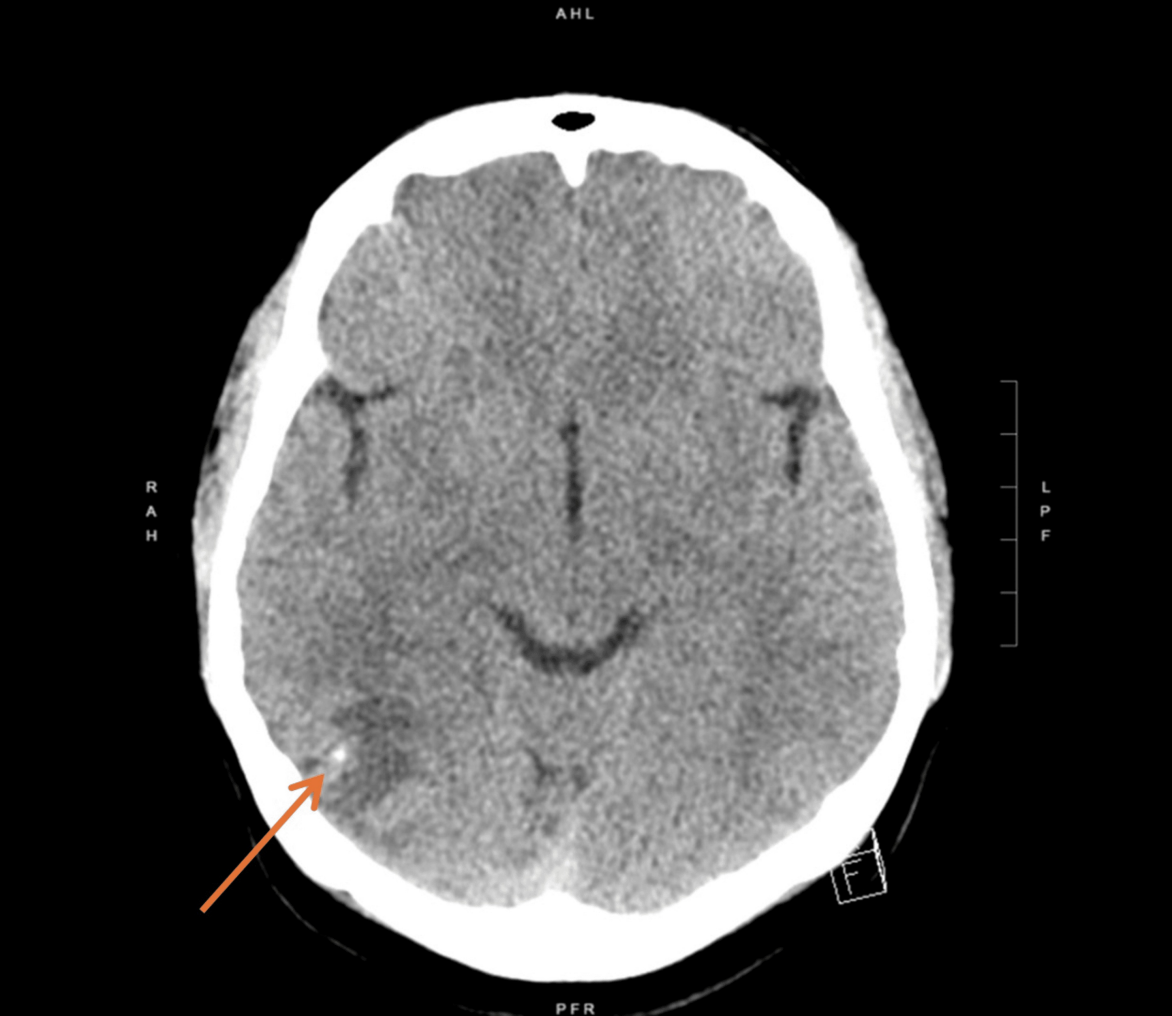
CT head without contrast scan showing a calcification in the right occipital lobe and vasogenic edema

Lab analyses were completed and were otherwise unremarkable (Table 1). A CT scan of her brain identified a small cortical calcific density of the right lateral occipital lobe with moderate subcortical hypoattenuation representing vasogenic edema (Figure 1).

**Table 1:** Emergency department laboratory analyses results WBC - white blood cells; RBC - red blood cells; Hb - hemoglobin; Hct - hematocrit; ESR - erythrocyte sedimentation rate; CRP - C-reactive protein; TSH - thyroid-stimulating hormone; BUN - blood urea nitrogen; ALT - alanine transaminase; AST - aspartate aminotransferase

Laboratory analysis	Result	Normal reference range
WBC	17.83 10*3/uL	3.7-10.3 10*3/uL
Neutrophils absolute	15.43 10*3/uL	1.6-6.10 10*3/uL
Lymphocytes absolute	1.45 10*3/uL	1.2-3.9 10*3/uL
Monocytes absolute	0.7 10*3/uL	0.3-0.9 10*3/uL
Eosinophils absolute	0.04 10*3/uL	0.0-0.5 10*3/uL
Basophil absolute	0.05 10*3/uL	0.0-0.10 10*3/uL
Immature granulocyte absolute	0.14 10*3/uL	0.00-0.06 10*3/uL
RBC	4.86 10*6/uL	3.9-5.2 10*6/uL 10*3/uL
Hb	14.6 g/dL	11.2-15.7 g/dL
Hct	41.5 %	34-45 %
Platelet count	273 10*3/uL	155-369 10*3/uL
Blood cultures	No growth day 5	
ESR	20 mm/hr	<20 mm/hr
CRP	<3.0 mg/dL	<8 mg/dL
Pregnancy test	Negative	
TSH	5.71 uIU/mL	0.4-5 uIU/mL
Glucose	115 mg/dL	74-99 mg/dL
Sodium	136 mmol/L	136-145 mmol/L
Potassium	4.0 mmol/L	3.6-4.9 mmol/L
Chloride	102 mmol/L	97-107 mmol/L
CO2	23 mmol/L	22-29 mmol/L
Creatinine	0.64 mg/dL	0.6-1.1 mg/dL
Anion gap	11 mmol/L	6-16 mmol/L
BUN	8 mg/dL	7-21 mg/dL
Calcium	9.4 mg/dL	8.9-10.2 mg/dL
Albumin	4.4 g/dL	3.5-5.2 g/dL
Total protein	8.2 g/dL	6.3-7.9 g/dL
ALT, plasma	19 U/L	10-35 U/L
AST, plasma	22 U/L	10-35 U/L
Total bilirubin, plasma	0.3 mg/dL	0.2-1.1 mg/dL
Alkaline phosphatase	64 U/L	35-104 U/L
Lactate	2.2 mmol/L	0.5-2.2 mmol/L

Neurosurgery and neurology were consulted in the emergency department. Neurosurgery recommended IV dexamethasone 10mg once and 4mg every six hours. They also recommended a stat CTA of her head for vascular lesions, which was unremarkable, and a stat magnetic resonance imaging (MRI) to be performed as an inpatient. Neurology recommended 1 gram of levetiracetam every 12 hours. The patient was admitted to the hospital medicine team for further workup of brain mass and new-onset seizures.

Inpatient course

**Figure 2:**
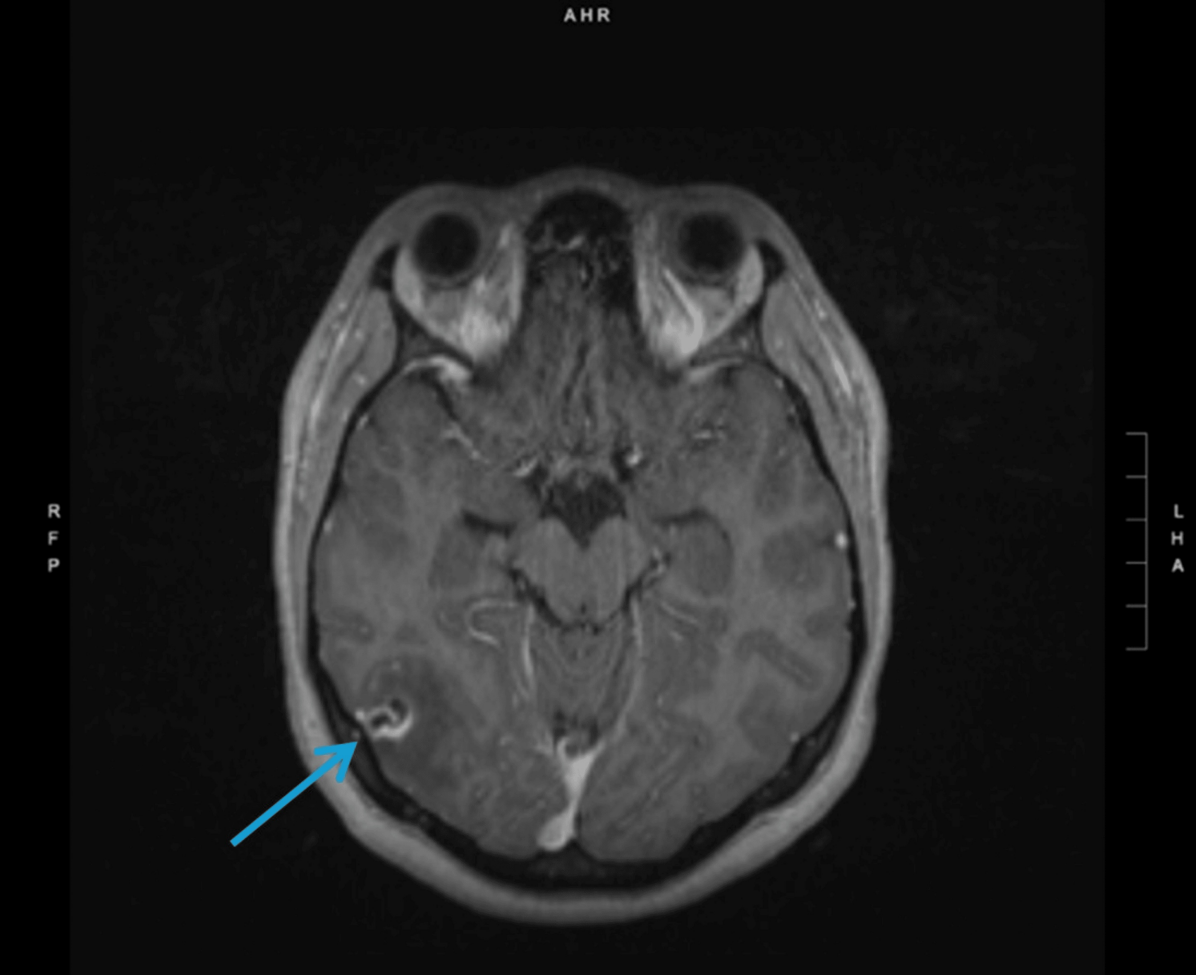
MRI showing a 12 mm ring-enhancing lesion with a central spot situated in the right lateral occipital lobe with surrounding edema

Once the patient was admitted, she underwent MRI imaging, which revealed a scolex: a solitary ring-enhancing lesion with punctate calcifications. The scolex was found in the right occipital lobe with surrounding vasogenic edema (Figure 2). 

Infectious disease (ID) was consulted and reported that the scolex seen on MRI was a pathognomonic finding for NCC. They recommended cysticercosis and strongyloides serology testing given her travel history and MRI findings. The serology was positive for cysticercosis (Table 2), which confirmed the diagnosis of NCC, and treatment was planned. Of note, the patient reported that her diet in Nepal mostly consisted of chicken and rice. However, she would consume pork approximately twice a month when she would eat out. 

**Table 2:** Results from infectious serology testing

Analysis	Results	Normal reference range
Cystercercosis antibody IgG	10 U	<8 U
Quantiferon tuberculosis	Negative	Negative
Strongyloides antibody IgG	0.3 IV	<0.9 IV
Parasite and ova exam from stool	Negative	Negative

ID recommended the standard treatment for NCC, which consisted of a regimen of albendazole 500mg twice daily for 14 days and a 21-day dexamethasone taper (for prevention of inflammation that can cause more seizures). Before initiation of treatment, ophthalmology was consulted to perform a fundoscopy exam. Ophthalmology findings were unremarkable for any intra-orbital parasites, and the treatment was initiated.

The patient did not have any further seizures or suffer any neurologic impairments while in the hospital. She was discharged after four days with recommendations of repeat MRI in six to eight weeks and a CBC in six to eight weeks for agranulocytosis screening secondary to albendazole use. She had follow-up appointments scheduled with infectious disease, neurology, and neurosurgery.

Outpatient course

The patient reported no further seizure-like activity at her follow-up appointments. She completed her course of anti-parasitics and steroids. Her eight-week repeat MRI showed resolution of the scolex lesion. She was advised to continue her anti-epileptics until cleared by neurology.

## Discussion

**Figure 3:**
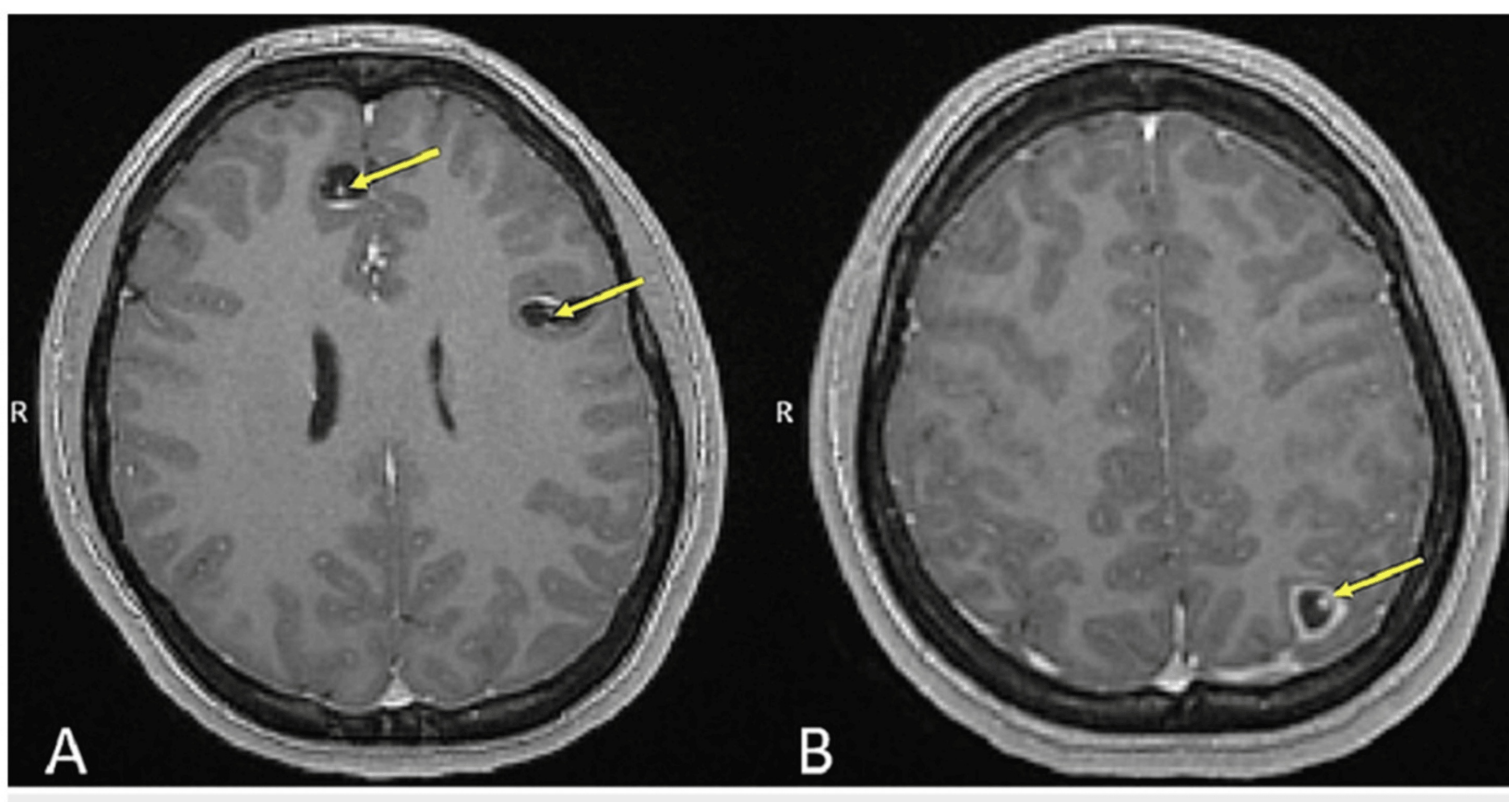
Scolex within the cysts seen on MRI Permission to use image granted by Dr. Norman Beatty [8]

Neurocysticercosis is a central nervous system (CNS) infection caused by the tapeworm Taenia solium, typically transmitted through the consumption of raw or undercooked pork [2,5]. The prevalence of NCC in Nepal is estimated at 0.002% to 0.1% [6]. After ingestion, the parasite's eggs can cross the intestinal barrier and enter the bloodstream, allowing them to travel throughout the body. These eggs may lodge in various tissues, where they develop into cysticerci-the larval form of T. solium. Each larva is a fluid-filled sac that uses a specialized structure called a scolex (the tapeworm's head) to anchor itself to host tissue. On imaging, the scolex often appears as a small, dense nodule within a cystic lesion and is pathognomonic for neurocysticercosis (Figure 3) [7,8]. When humans ingest infected pork meat, the scolex attaches to the human's intestinal wall and develops into an adult tapeworm that can grow to the size of 2-4m and produces eggs that are excreted in the feces [2,6].

NCC is most commonly found in developing countries in South America, Africa, and Asia, and is a major cause of adult-onset epilepsy and a prevalent cause of neurological hospital admissions in the developing world [2]. Cases of NCC in developed countries such as the United States are usually found in people traveling from these endemic areas.

Cysticercosis can be found in any organ, but when the cysts are in the CNS, they present as seizures [2]. NCC can be diagnosed using the Del Brutto Criteria (Table 3) [8]. The patient in this case was diagnosed using an absolute criterion: evidence of cystic lesions showing the scolex on neuroimaging studies. Her diagnosis was supported by positive serologic studies and travel to an endemic area [9]. 

**Table 3:** Del Brutto Diagnostic Criteria for Neurocysticercosis Del Brutto Diagnostic Criteria for Neurocysticercosis [8]

Del Brutto Diagnostic Criteria for Neurocysticercosis	
Absolute criteria	Histological demonstration of the parasite from biopsy of a brain or spinal cord lesion
	Evidence of cystic lesions showing the scolex on neuroimaging studies
	Direct visualization of subretinal parasites by fundoscopic examination
Major criteria	Evidence of lesions highly suggestive of neurocysticercosis on neuroimaging studies
	Positive serum immunoblot for the detection of anticysticercal antibodies
	Resolution of intracranial cystic lesions after therapy with albendazole or praziquantel
	Spontaneous resolution of small single enhancing lesions
Minor criteria	Evidence of lesions compatible with neurocysticercosis on neuroimaging studies
	Presence of clinical manifestations suggestive of neurocysticercosis
	Positive cerebrospinal fluid ELISA for detection of anticysticercal antibodies or cysticercal antigens
	Evidence of cysticercosis outside the central nervous system
Epidemiological criteria	Individuals coming from or living in an area where cysticercosis is endemic
	History of travel to disease-endemic areas
	Evidence of a household contact with Taenia solium infection
Degrees of diagnostic certainty	
Definitive	Presence of one absolute criterion
	Presence of two major plus one minor and one epidemiological criteria
Probable	Presence of one major plus two minor criteria
	Presence of one major plus one minor and one epidemiological criteria
	Presence of three minor plus one epidemiological criteria

The most effective way to decrease the incidence of NCC is through prevention by cooking pork to an internal temperature of 145° Fahrenheit before eating and increasing education and awareness of the disease [10]. When cysts are present in the brain, treatment focuses on eliminating the parasite and managing the inflammation responsible for seizures. To prevent complications of vision loss, a fundoscopic exam must be completed to ensure there are no cysts in the eye prior to treatment [11]. The treatment regimen typically includes: a corticosteroid, such as a 21-day tapering course of dexamethasone, to reduce inflammation secondary to cyst degeneration; an anti-epileptic medication, usually levetiracetam, administered for at least six months to control seizures, 24 months for multiple lesions; an antiparasitic therapy, consisting of a 14-day course of albendazole, with the addition of praziquantel if more than two cysts are present. Monitor for agranulocytosis, which can be seen with long-term use of albendazole. Of note, patients who need prolonged corticosteroids should also be tested for tuberculosis (TB) and strongyloidiasis. Neuroimaging must be repeated every six months after the antiparasitic therapy until the cysts resolve. If the lesions persist for more than six months, the patient will need another course of antiparasitic therapy.

## Conclusions

Neurocysticercosis (NCC) is a common disease in developing countries, but its incidence is rising in the United States. Timely recognition and treatment are critical, as NCC is the leading cause of adult-onset epilepsy worldwide. Prevention remains the most effective strategy, primarily through proper food handling. Diagnosis involves a combination of neuroimaging, including CT and MRI scans, along with serologic testing. Treatment typically includes antiseizure medications such as levetiracetam, antiparasitic therapy with albendazole, and corticosteroids to reduce inflammation. Importantly, before starting antiparasitic treatment, a fundoscopy must be performed by ophthalmology to rule out ocular cysts and avoid complications such as vision loss.
